# Estimation of the stress related to conservative scoliosis therapy: an analysis based on BSSQ questionnaires

**DOI:** 10.1186/1748-7161-2-1

**Published:** 2007-01-03

**Authors:** Tomasz Kotwicki, Edyta Kinel, Wanda Stryła, Andrzej Szulc

**Affiliations:** 1 Department of Pediatric Orthopedics and Traumatology, University of Medical Sciences of Poznan, ul. 28 Czerwca 1956 roku nr 135, 61-545 Poznan, Poland; 2 Department of Rehabilitation, University of Medical Sciences of Poznan ul. 28 Czerwca 1956 roku nr 135, 61-545 Poznan, Poland

## Abstract

**Background:**

Adolescent girls treated with a brace for scoliosis are submitted to prolonged stress related to both the disease and the therapy. Currently proposed quality of life questionnaires are focused on the outcome of therapy. Bad Sobernheim Stress Questionnaire (BSSQ) enables monitoring of patients being under treatment with a brace or exercises. The aim of the study was to assess the stress level in conservatively managed scoliotic girls using BSSQ.

**Materials and methods:**

111 girls, aged 14,2 ± 2,2 years, mean Cobb angle of the primary curve 42,8° ± 17,0° and mean Bunnell angle of 11,4° ± 4,5° were examined with two versions of BSSQ (Deformity and Brace). The analysis considered the type of treatment, curve location, correlation of the total score with age, Cobb angle and Bunnell rotation angle.

**Results:**

The BSSQ Deformity revealed the median of 17 points in patients managed with exercises (from 4 to 24 points), 18 in patients managed with a brace (from 8 to 24 points) and 12 in patients before surgery (from 3 to 21 points). Braced patients who completed both questionnaires (n = 50) revealed significantly higher score with BSSQ Deformity (median = 18) comparing to BSSQ Brace (median = 9). There was a correlation between the total score of BSSQ Deformity and the Cobb angle (r = -0,34), Bunnell primary curve rotation (r = -0,34) and Bunnell sum of rotation (r = -0,33) but not with the age of patients.

**Conclusion:**

Scoliotic adolescents managed with exercises and brace suffered little stress from the deformity. The brace increased the level of stress over the stress induced by the deformity. The stress level correlated with clinical deformity (Bunnell angle), radiological deformity (Cobb angle) and the type of treatment (exercises, bracing, surgery). Bad Sobernheim Stress Questionnaires are simple and helpful in the management of girls treated conservatively for idiopathic scoliosis.

## Background

The quality of life of patients suffering from idiopathic scoliosis and submitted to various treatments is the object of increasing interest of the professionals. Questionnaires assessing the quality of life are applied to patients who have already completed their treatment. SRS-22 [1] and SF-36 [2] seem the most diffused. A continuous process of improving the existing questionnaires can be observed. However the other questionnaires address more specifically the situation of the patient currently being under the treatment [3].

The treatment of a child or an adolescent with idiopathic scoliosis often lasts months or even years and requires positive mobilization of the patient and her parents. Not only the disease which is producing a visible deformity of the body, but also the treatment itself may stress the patient and induce stress reactions. Wearing a corrective brace at school or outside home is the reason for both physical and psychological discomfort. There is still an insufficient level of acceptance of such a treatment at school society. One can only wonder why a dental corrective device is approved by the children, or even considered trendy, while a spinal corrective device is not.

There is a need for monitoring the level of stress in patients managed conservatively for progressive scoliosis. Weiss et al. constructed two questionnaires designated to assess the stress induced by the deformity (Bad Sobernheim Stress Questionnaire Deformity, BSSQ-Deformity) as well as by the treatment with a brace (Bad Sobernheim Stress Questionnaire Brace, BSSQ-Brace), [4,5]. We decided to apply the questionnaires to patients treated at our Institution. The aim of the study was to analyze our short term experience with the BSSQ.

## Materials and methods

We examined 111 consecutive patients, girls, aged from 10 to 20 years, mean 14,2 ± 2,2 years. The Cobb angle of the primary curve varied from 20° to 92°, mean 42,8° ± 17,0°. The Bunnell scoliometer angle of trunk rotation (ATR)[6] of the main curve varied from 5° to 30°, mean 11,4° ± 4,5°. The sum of ATRs measured at three different levels of the back (upper thoracic, main thoracic and lumbar) varied from 8° to 43°, mean 18,6° ± 7,2°.

**Figure 1:**
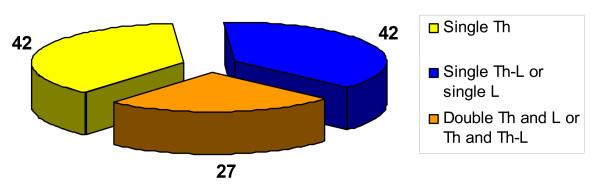
Distribution of patients according to the type of the curve. The single curves were analyzed separately for the thoracic and lumbar location. Double curves were grouped together.

**Figure 2:**
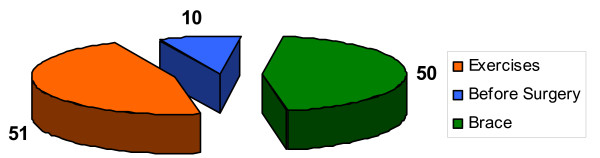
Distribution of patients according to the type of management. Braced patients received specific physiotherapy. Exercise managed patients received exercises of various types, often unspecific, out of our Institution.

Patients were classified according to the location of the curve: 1) single primary thoracic curve – 42 patients (38%), 2) single primary thoraco-lumbar or lumbar curve – 27 patients (24%), and 3) double primary thoracic and lumbar or double primary thoracic and thoraco-lumbar curve – 42 patients (38%), figure 1. The patients were managed with: 1) physiotherapy – 51 patients (46%), 2) bracing and physiotherapy – 50 patients (45%), 3) preparation to surgical treatment – 10 (9%), figure 2. All the braced patients had the same type of brace (Cheneau), manufactured by the team from our Institution.

The BSSQ Deformity and the BSSQ Brace questionnaires contain eight questions each. Two questions serve to test the plausibility. There are four possible answers to each question and the number of points from 0 to 3 is attributed for each answer. The sum of all answers indicates the total score of the questionnaire. The score ranges from zero to twenty four points. The score correlates negatively with the stress level (more points indicate less stress). The minimal value of zero points indicates maximal stress and the maximal value of 24 points indicates minimal stress. The range 0 to 8 points denotes strong stress, 9 to 16 denotes medium stress, 17 to 24 denotes little stress.

All the patients were examined with the BSSQ-Deformity. The brace treated patients were additionally examined with the BSSQ-Brace. The score of the patients managed with exercises and examined with the BSSQ-Deformity was compared to the score of the patients managed with a brace and examined with the BSSQ-Brace. The score of the patients measured with the BSSQ-Deformity was analyzed in the three subgroups separately: the braced patients, the physiotherapy patients and the patients prepared to scoliosis surgery. The patients managed with a brace were asked to fill both questionnaires and the score of each one was analyzed. The time of wearing the brace was noted. The braced patients were classified as: 1) full time braced (20 hours per 24), 2) part time (out of school, 12–16 hours per 24), 3) nighttime braced (8 hours per 24). The median, minimum and maximum of the total score were taken. The correlation of the total score with the age, Cobb angle, Bunnell primary curve rotation and Bunnell sum of rotation was checked. The BSSQ Deformity value was analyzed separately for the group of age 10 to 14 years and for the age 15 to 17 years. P value of 0,05 was considered significant.

## Results

All the girls answered all the questions. No patients reported difficulties in understanding any question. The time of the exam did not exceed 5 minutes per questionnaire. For the BSSQ Deformity the maximal value of 24 points was achieved by 10 patients (9,0 %), no patient achieved the total score of 0, 1 or 2 points, one patient scored 3 points and 11 patients (10,0 %) scored below 10 points. For the BSSQ Brace the maximal value of 24 points was not achieved, one patient scored 21 points, 25 patients (50 %) scored below 10 points and one patient scored the minimal value of 0 points. The test for the plausibility in the BSSQ-Deformity revealed 57 patients fulfilling the criteria and 54 not fulfilling. The analysis made for patients examined exclusively with the BSSQ-Deformity revealed 29 patients fulfilling the criteria and 28 not fulfilling. In the group of patients treated with a brace 25 fulfilled the plausibility and 29 did not.

**Figure 3:**
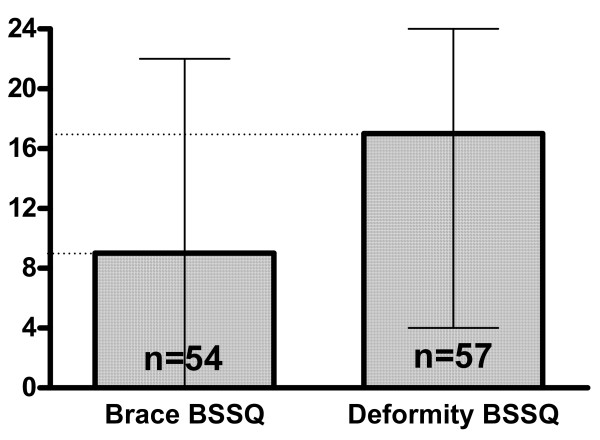
The median of the score of the questionnaire: (a) the patients treated with a brace, examined with the BSSQ-Brace, (b) the patients managed with exercises, examined with the BSSQ-Deformity. The ten patients admitted for spinal surgery were divided into groups (a) or (b), depending on whether they previously had brace treatment or not. The squares indicate the value of the median; the slim lines indicate the minimum and maximum.

**Figure 4:**
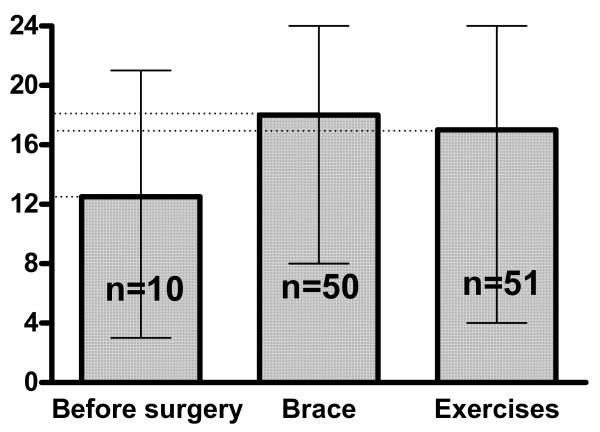
The median of the score achieved with the BSSQ-Deformity by the patients admitted for surgical correction of scoliosis (a), treated with a brace (b) or managed with exercises (c). The squares indicate the value of the median; the slim lines indicate the minimum and maximum.

**Figure 5:**
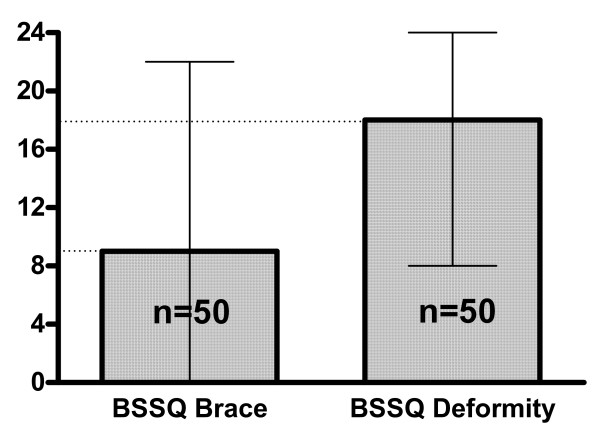
The scores of two questionnaires: BSSQ-Brace and BSSQ-Deformity applied to the same population of patients treated with a brace. The squares indicate the value of the median; the slim lines indicate the minimum and maximum. Difference highly significant. The patients reveal more stress because of the orthosis than because of the disease.

There was a significant difference between the score in patients managed with exercises and examined with BSSQ Deformity (median = 17), comparing to patients managed with a brace and examined with BSSQ Brace (median = 9), figure 3. The BSSQ Deformity revealed the median of 17 points in patients managed with exercises, 18 in patients managed with a brace and 12 in patients before spinal surgery, figure 4. Braced patients who were asked to complete both questionnaires (n = 50) revealed significantly higher score with BSSQ Deformity (median = 18) comparing to BSSQ Brace (median = 9), figure 5.

**Figure 6:**
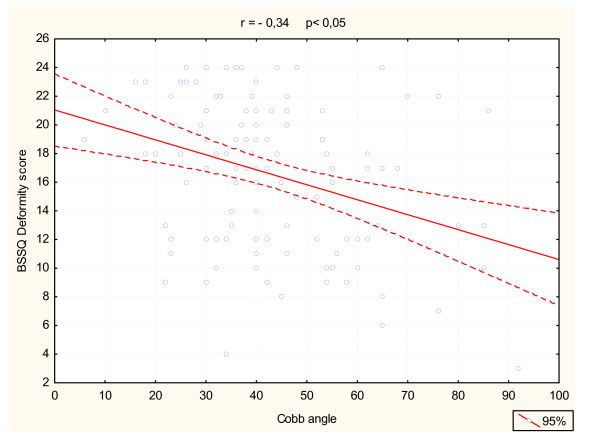
Correlation of BSSQ Deformity total score and Cobb angle.

There was a correlation between the total score of BSSQ Deformity and the following parameters: Cobb angle (r = -0,34, p < 0,05, figure 6), Bunnell primary curve rotation (r = -0,34, p < 0,05) and Bunnell sum of rotation (r = -0,33, p < 0,05). There was no correlation between the BSSQ-Deformity score and the age of patients. There was no significant difference at the stress level measured with BSSQ Deformity between the group of 10 to 14 years of age and the group of 15 to 17 years of age (Mann-Whitney U test, p = 0,816). Time of brace wearing did not influence the total BSSQ-Deformity score significantly. For the BSSQ Brace the median of the night-time braced patients was 7, the part time braced patients was 9 and the full time braced was 12, the tendency of getting more stressed when wearing the brace shorter, but not significant (ANOVA).

## Discussion

Both questionnaires were understandable for girls of all ages and there was apparently no difficulty for the patients to answer all the questions. There might be a doubt at this point as the test of plausibility revealed as much as 54 patients not fulfilling the criteria. We think that the construction of questions 2, 3 and 4 could be revised for coherence in the plausibility test. The minimal value was scored once (BSSQ Brace) by a girl, examined at her first visit, just after she learned about brace necessity. The treating regime was modified for this girl to allow her a longer period of adaptation to the brace treatment and a good compliance was finally reached. There was a tendency to get higher scores with BSSQ Deformity than with the BSSQ Brace. The analysis of five patients with the lowest results of the BSSQ Brace (3–5 points) revealed a characteristic profile: the girls who had poor communication with their parents. Two of them refused the brace, progressed and were admitted for surgery. The surgical patients presented the lowest scores related to their high grade of deformity.

The score of the BSSQ Deformity was equal for the brace treated patients compared to the exercise treated patients (figure 4). The braced patients revealed more stress when investigated for their braces than for their deformity (figure 5). This finding demonstrates that the brace treatment is a difficult task for the patient. The responsibility of the treating physician is to apply such a treatment in a carefully balanced manner. On the other hand the decreased quality of life during brace treatment may be considered a transient phenomenon. After completion of therapy one can expect the improvement of the patient's emotional state.

The relatively moderate but significant correlation of the BSSQ score with Cobb angle and Bunnell angle indicates that the stress level directly depends on the objectively measured parameters, describing the severity of the disease, not as an exclusive factor but in inter-dependence with other variables. The influence of the patient's personality, family and school environment, or physical activity could not be analyzed in this study. As the correlation between the BSSQ score and the patients' age was not found, further analysis was performed by dividing the patients into two age subgroups: 10 to 14 years (n = 62) and 15 to 17 years (n = 42). From the psychosocial point of view during the first period adolescents are particularly sensitive about body defects. During the second period they want to make the body appear more attractive. No significant difference in the stress level between the two subgroups was found. The girls of 18 years of age or older were not analyzed as a separate subgroup because of the small sample size (n = 7).

The majority of questionnaires used for patients with idiopathic scoliosis are constructed with the goal of the estimation of the final outcome of the treatment. We found the BSSQ to be helpful for determining the level of stress during scoliosis therapy. It is generally recognized that the compliance is one of the paramount factors for the correct course of the non-operative scoliosis treatment [7]. A good interpersonal relation between the patients and the treating team influences the final result. In our opinion the advantage of the continuous monitoring of the stress level concerns the possibility of modification of the treatment in order to maintain good compliance. Thus the BSSQs are helpful tools in conservative scoliosis therapy.

## Conclusion

1. Scoliotic adolescents managed with exercises and brace suffered little stress from the deformity.

2. The brace increased the level of stress over the stress induced by the deformity.

3. The stress level correlated with clinical deformity (Bunnell angle), radiological deformity (Cobb angle) and the type of treatment (exercises, bracing, surgery).

4. Bad Sobernheim Stress Questionnaires are simple and helpful in the management of girls treated conservatively for idiopathic scoliosis.

## Competing interests

The author(s) declare that they have no competing interests.

## Authors' contributions

TK and EK participated in the concept and design of the study, data acquisition and analysis. WS and AS supervised the study and helped to draft the manuscript. All authors read and approved the final manuscript.
